# Lactate metabolism and protein lactylation in cancer

**DOI:** 10.1186/s43556-026-00417-4

**Published:** 2026-02-26

**Authors:** Zhaoyun Liu, Aili Li, Ziyu Ma, Junzhu Wang, Xinyu Chen, Zhiwei Wang, Rong Fu

**Affiliations:** Department of Hematology, Tianjin Medical University General Hospital, Tianjin Key Laboratory of Bone Marrow Failure and Malignant Hemopoietic Clone Control, Tianjin Institute of Hematology, State Key Laboratory of Experimental Hematology, 154 Anshan Street, Heping District, Tianjin, 300052 China

**Keywords:** Lactylation, Histone modification, Tumor microenvironment, Immune evasion, Cancer therapy, LDH

## Abstract

Lactylation is a recently identified post-translational modification that links cellular metabolism to gene regulation, playing pivotal roles in cancer development and the tumor microenvironment (TME). Derived from lactate produced by glycolysis and glutamine metabolism, lactylation occurs on both histone and non-histone proteins, modulating transcription, protein function, and cellular signaling. In tumors, lactylation contributes to proliferation, metastasis, therapy resistance, and immune evasion by influencing the function of Treg cells, macrophages, dendritic cells, and NK cells. Its dynamic regulation by “writers” (e.g., p300), “erasers” (e.g., Histone deacetylases (HDACs), Sirtuins3 (SIRT3)), and transporters (e.g., monocarboxylate transporters (MCT) 1/4) provides multiple intervention points for therapy. Preclinical studies demonstrate that targeting lactylation directly or indirectly—through LDH (lactate dehydrogenase) inhibition, MCT blockade, or modulation of lactyltransferases—enhances the efficacy of immune checkpoint inhibitors, Chimeric Antigen Receptor T (CAR-T) therapy, and chemotherapeutic agents.Despite these advances, critical questions remain regarding the specificity of lactylation compared with other post-translational modifications, the tumor types most dependent on lactylation, and reliable biomarkers to guide treatment. Additionally, clinical validation of lactylation-targeting strategies is limited. Future research integrating mechanistic studies, patient-derived samples, and multi-omics approaches is essential to elucidate context-dependent functions, refine therapeutic targets, and develop precision interventions.This review provides a comprehensive summary of lactylation biology in cancer, highlighting its metabolic-epigenetic interplay, immunomodulatory roles, and therapeutic potential. By synthesizing current evidence, we aim to guide future studies and clinical strategies targeting lactylation to improve cancer treatment outcomes.

## Introduction

Cellular metabolism plays a central role in maintaining energy homeostasis and regulating diverse biological processes. Lactate, long considered a metabolic waste product generated through glycolysis, has recently been recognized as a critical signaling molecule that modulates gene expression, immune responses, and tumor progression [1]. Early studies, including Warburg’s observation of aerobic glycolysis in tumor cells, revealed that lactate accumulates in the tumor microenvironment (TME), influencing both cancer and stromal cells [2, 3]. Beyond its metabolic role, lactate serves as a substrate for lysine lactylation (Kla), a novel post-translational modification, linking cellular metabolism directly to epigenetic regulation [1, 4]. This discovery has expanded our understanding of how metabolic rewiring contributes to cancer biology and immune evasion.

Lactylation occurs on histone and non-histone proteins, affecting transcription, protein activity, and signaling pathways [1, 5]. In tumors, histone Kla influences gene expression programs that promote proliferation, metastasis, angiogenesis, and therapy resistance [6, 7]. Non-histone lactylation modulates enzyme activity, immune cell function, and tumor-stroma interactions. Importantly, lactylation is dynamically regulated by enzymes (“writers,” “erasers”) and transporters, such as p300, HDACs, SIRT family proteins, and monocarboxylate transporters (MCT1/4), which serve as potential therapeutic targets [1]. Preclinical evidence indicates that targeting lactylation directly or indirectly can enhance the efficacy of immunotherapies, CAR-T therapy, and conventional chemotherapy, highlighting its translational potential [8–12].

Despite these advances, key questions remain unresolved. The specificity of lactylation relative to other post-translational modifications, the tumor types most dependent on lactylation, and the availability of reliable biomarkers for guiding therapy are not fully understood. Moreover, clinical evidence for lactylation-targeting strategies is limited, underscoring the need for mechanistic studies and patient-derived validation. A systematic synthesis of current research is essential to identify gaps, evaluate therapeutic potential, and guide future investigations.

**Fig. 1:**
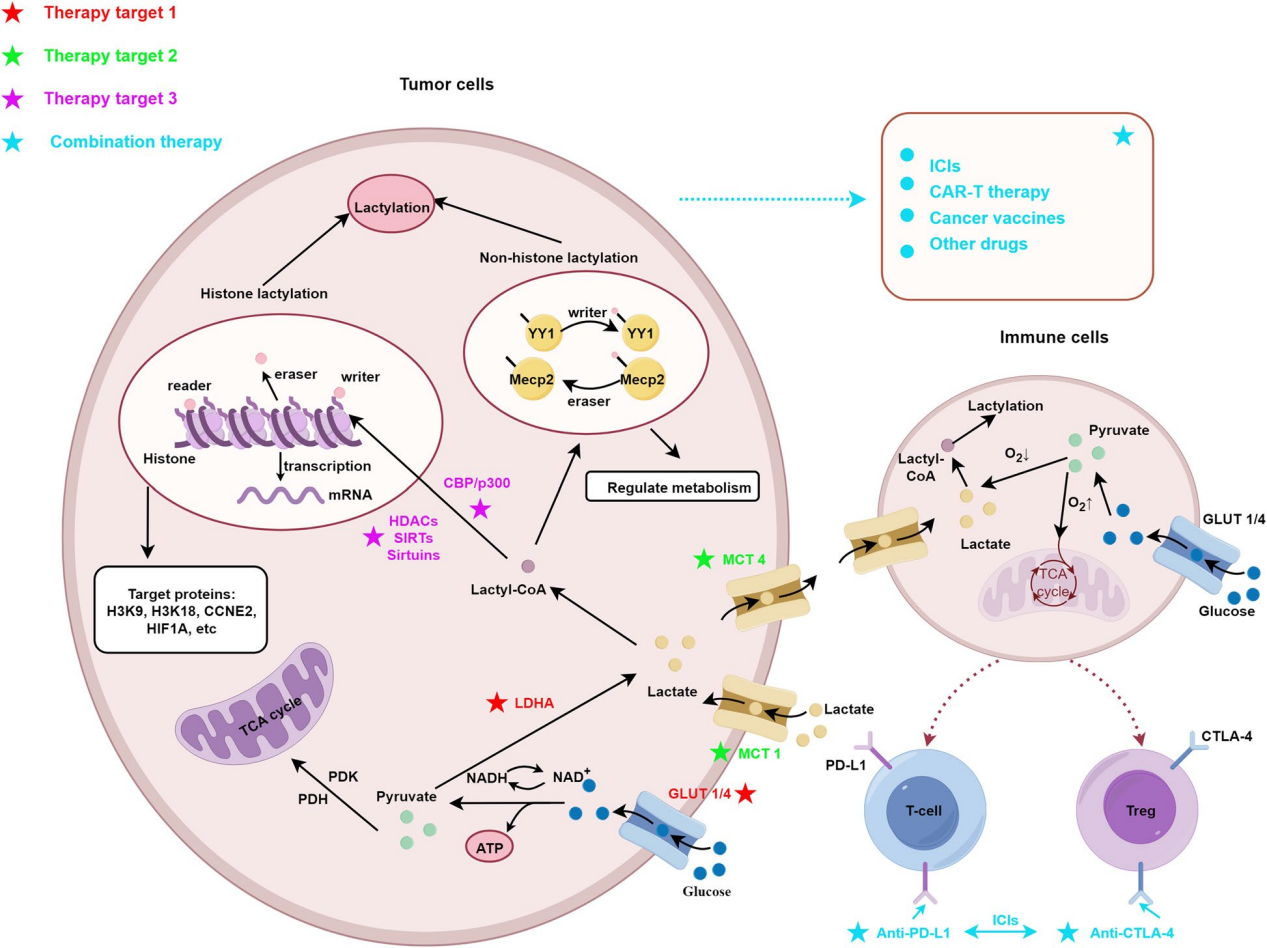
This schematic illustrates the mechanisms of lactylation-mediated crosstalk between tumor cells and immune cells, along with potential therapeutic targets and combination therapies. In tumor cells, lactylation (histone and non-histone) regulates transcription and metabolism, involving key molecules like LDHA, GLUT1/4, MCT1/4, and modifiers (writers, erasers, readers). Lactate produced by tumor cells affects immune cell function (T cells, Treg cells) via metabolic pathways and immune checkpoints (PD-L1, CTLA-4). Therapeutic targets are categorized by color-coded stars, and combination therapies include ICIs, CAR-T, cancer vaccines, and other drugs, highlighting strategies to disrupt lactylation-driven tumor immune evasion. Abbreviations: LDHA, lactate dehydrogenase A; GLUT1/4, glucose transporter 1/4; MCT1/4, monocarboxylate transporter 1/4**;** Treg cells, regulatory T cells; PD-L1, programmed death-ligand 1; CTLA-4, cytotoxic T-lymphocyte-associated protein 4; ICIs, immune checkpoint inhibitors; CAR-T, chimeric antigen receptor T-cell immunotherapy

This review comprehensively summarizes the biology of lactylation in cancer, with a focus on its metabolic-epigenetic interplay, immunomodulatory roles, and potential as a therapeutic target. We first describe lactate metabolism and the mechanisms of histone and non-histone lactylation. Next, we discuss the effects of lactylation on tumor progression, immune cell regulation, and therapy resistance. Finally, we highlight preclinical and emerging clinical strategies targeting lactylation, providing a roadmap for future research and clinical translation. The summary diagram of this article is shown in Fig. 1.

## Lactate metabolism and lactylation

Lactate metabolism plays a central role in both normal physiology and tumor biology, linking energy production, metabolic reprogramming, and cellular signaling. In this section, we summarize the pathways of lactate production, transport, and the emerging role of lactylation as a post-translational modification that integrates metabolic signals with gene regulation.

### Lactic acid production and transport

Lactate metabolism is a central process in tumor biology, linking energy production with metabolic signaling and protein lactylation. In this section, we summarize how lactate is produced in cancer cells, transported across membranes, and functions as a substrate for lactylation-mediated regulation.

Lactylation has recently emerged as a novel form of protein modification. Historically, lactate was regarded as a metabolic waste product generated during glycolysis under hypoxic conditions [13, 14]. However, Warburg found that tumor cells also undergo anaerobic glycolysis and secrete large amounts of lactate, even in the presence of sufficient oxygen, a form of cellular metabolism known as the Warburg effect [2, 15, 16]. Tumor cells can also produce lactic acid through glutamine catabolism. Although the energy generated via glycolysis and glutamine catabolism is smaller than that produced via aerobic oxidation, this metabolic shift is advantageous for tumor cell proliferation [3, 17, 18].

Receptors are involved in intercellular lactate transport. Furthermore, intracellular and extracellular lactate transport is mainly mediated by MCT1 and MCT4 [4]; lactate uptake is mediated by MCT1, whereas lactate output is mediated by MCT4 [19]. Other receptors that can bind to lactate also exist in the cell. G protein-coupled receptors, such as GPR81, which is a lactate receptor located on the cell membrane that can act as a signaling molecule and can be directly manipulated intracellularly in response to extracellular lactate stimulation via multiple pathways [20]. In the nervous system, lactate can bind to the receptor for hydroxycarboxylic acid receptor 1 (HCAR1) and initiate reparative effects [21].

**Fig. 2:**
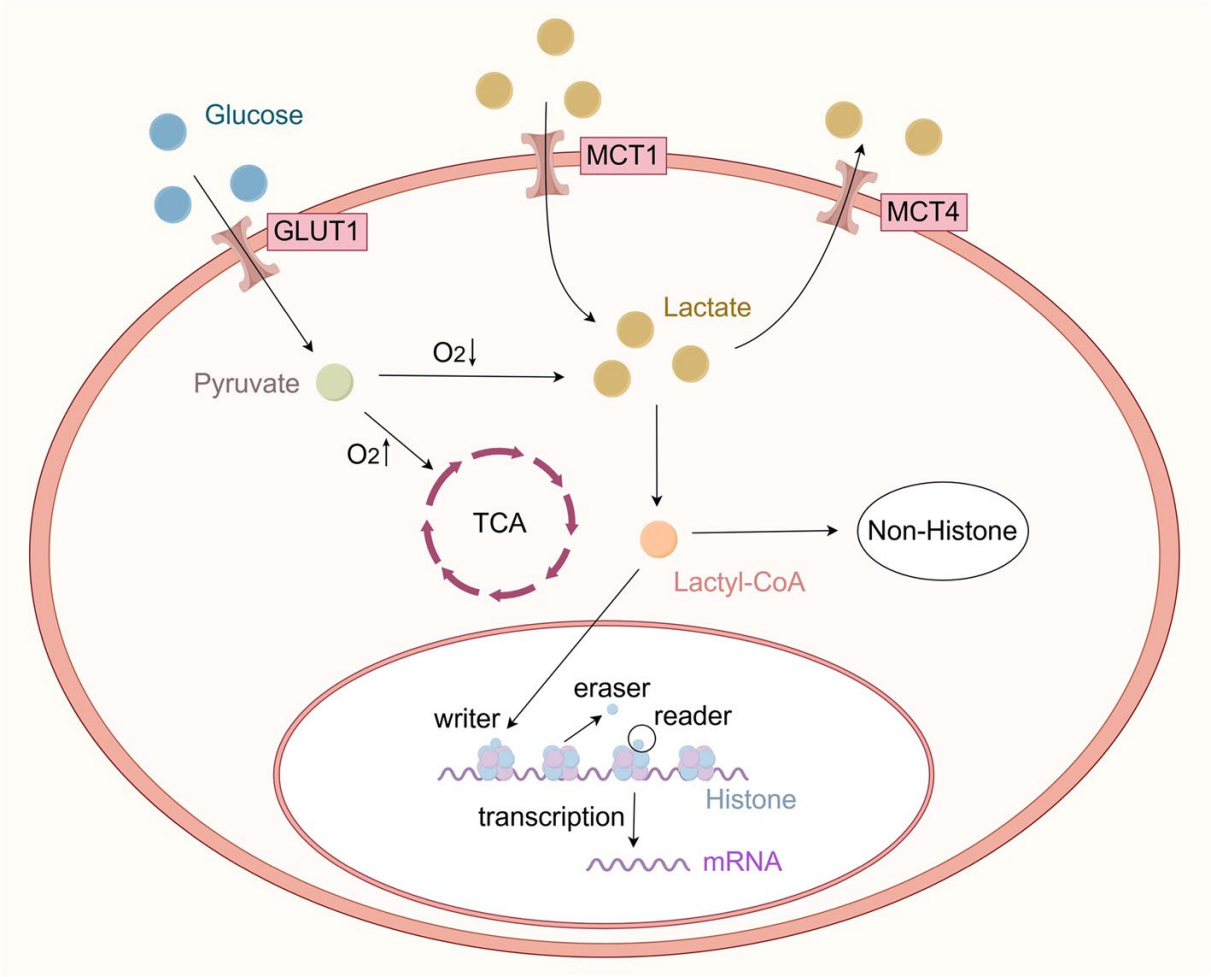
Schematic diagram of lactate metabolism. Tumor cells undergo anaerobic glycolysis and secrete large amounts of lactic acid, even in the presence of sufficient oxygen, a form of cellular metabolism known as the Warburg effect. Combined with the processing of raw material requirements, this approach is more conducive for tumor cell proliferation than aerobic oxidation. Lactate uptake is mediated by monocarboxylate transporter 1 (MCT1), while lactate output is mediated by MCT4. Histones, which are the major components of nucleosomes, can be regulated through various covalent modifications, which can affect gene transcription. Lactylation sites do not occur exclusively with histones; it can also occur with nonhistone proteins. Nonhistone proteins have significantly more sites than histones. Lactylation sites play a role in gene transcription, DNA damage repair, cell division, signaling, protein folding, autophagy, and metabolism via protein structure modification. Lactylation modifications are not irreversible but rather occur in a state of dynamic equilibrium. This dynamic equilibrium is mainly regulated by a lactosyltransferase “writer” and a delactonase “eraser,” which are responsible for the addition and removal of lactide groups, respectively. In turn, these enzymes are read and recognized by the “reader.” Abbreviations: MCT1/4, monocarboxylate transporter 1/4

Collectively, these mechanisms highlight lactate not only as a metabolic intermediate but also as a signaling molecule that influences protein lactylation and downstream cellular functions. Understanding lactate production and transport provides the foundation for exploring the regulatory roles of lactylation in tumor progression (Fig. 2).

### Lactylation

Histones, which are precursory components of nucleosomes, can be regulated through various covalent modifications that affect gene transcription [22]. Lactylation, a novel post-translational modification, has recently emerged as an important mechanism capable of modifying histones as well. Lactylation sites are integral to the disease mechanism affecting protein structure and function and may contribute to gene transcription, DNA damage repair, cell division, signal transduction, protein folding, autophagy, and metabolism [6, 7, 23]. However, lactylation is not limited to histones; it can also modify non-histone proteins [5, 24–31]. In fact, nonhistone proteins have been shown to have significantly more lactylation sites than histones, which suggests a broader functional role beyond epigenetic regulation [5, 32, 33]. The specific mechanism is shown in Fig. 1. However, the clinical relevance of nonhistone lactylation is still under investigation and requires further validation in human models.

#### Histone lactylation

Histone Kla, first identified by Zhang et al. as a metabolite-derived post-translational modification, forms via the binding of lactyl groups to histone lysine residues and is detectable by LC–MS/MS [1]. Its level is directly linked to cellular lactate content: enhanced glycolysis (e.g., hypoxia, glucose supplementation) promotes the accumulation of this modification, while inhibiting lactate production results in its reduction [1, 34]. To date, 28 Kla sites have been identified in the core histones of humans and mice; certain sites, such as H3K18la and H3K23la, display tissue-specific functions. For example, H3K18la has been shown to play a role in endometrial remodeling and transcriptional regulation in embryonic stem cells and may serve as a potential biomarker for septic shock, though its clinical utility has yet to be confirmed in large-scale studies [1, 35–38].

In terms of regulation, histone Kla is catalyzed by the HAT p300 and removed by enzymes like HDAC1–3 and SIRT1–3; lactate transport via MCTs also plays a key role in modulating its intracellular levels [1, 39–43]. Physiologically, Kla is involved in various biological processes, including embryonic development, osteoblast differentiation (e.g., H3K18la regulates JunB expression to support bone formation), and neural transcriptome remodeling [35, 38, 44, 45]. Pathologically, it is associated with multiple conditions, such as inflammation (e.g., activation of reparative genes post-myocardial infarction), tumors (e.g., H4K12la drives anaplastic thyroid cancer proliferation), and Alzheimer’s disease (activation of glycolytic genes in AD microglia) [29, 46, 47]. These findings have been observed in animal models, but further clinical studies are necessary to establish their relevance in human disease.

#### Non-histone lactylation

Non-histone Kla is widely present in various proteins, and its site count significantly exceeds that of histone Kla in certain contexts, such as hepatocellular carcinoma [5, 48, 49]. This modification primarily affects cellular processes by regulating protein structure and activity, rather than solely engaging in epigenetic regulation, as observed in multiple studies [5, 32]. Identified targets include a variety of protein classes: immune-related HMGB1, which triggers endothelial dysfunction after Kla modification [50]; Kla of transcription factors (e.g., YY1, HIF1α), which promotes angiogenesis and stabilizes proteins that support prostate cancer progression [49–51]; and Kla of glycolytic enzymes (e.g., ALDOA, α-enolase), which inhibits enzymatic activity or disrupts substrate binding, forming a feedback loop for lactate regulation [52]. While these findings are promising, their clinical implications remain speculative and require further validation through clinical trials.

In terms of regulation, non-histone Kla shares some enzymes with histone Kla (e.g., SIRT3 reverses the pro-tumor Kla modification of CCNE2), while prokaryotes utilize specific regulatory enzymes like YiaC and CobB [40, 53]. Functionally, non-histone Kla not only affects transcription and metabolism but is also involved in immune regulation (e.g., Kla of MOESIN modulates Treg cell function), autophagy (Kla of Vps34 promotes autophagosome maturation), and tumor metastasis (e.g., Kla of NUSAP1 drives pancreatic cancer progression), serving as a key mechanism that integrates metabolism and cellular function [32, 54–57]. These mechanisms have been demonstrated in preclinical models, but their direct relevance to human cancer remains speculative and requires confirmation in clinical trials.

### “Writer” and “eraser” in lactic acid modification

Lactylation is not irreversible but occurs in a state of dynamic equilibrium. This dynamic equilibrium is mainly regulated by a specific lactosyltransferase “writer” and a delactonase “eraser”, which are responsible for the addition and removal of lactide groups, respectively [1, 23, 34, 58]. In addition, these enzymes are read and recognized by a “reader” [23, 59]. p300 was identified not only as a histone acetyltransferase but also as a lactosyltransferase [60]. Both p300 and other writers catalyze many types of protein modifications that attach L-lactate to histones. The histone deacetylases (HDAC) 1 to 3 and SIRT1-3 can catalyze the removal of lactoyl groups, indicating their delactonization ability [13, 38, 61]. Recently one study showed that ATAT1 also serves as a lactylation writer for NAT10 [62]. Although these findings are significant, clinical studies are still needed to confirm the implications of this reversible process in human diseases.

Among the imbalances between the writer and eraser, an overactive writer will most likely induce the development of various lesions. This finding not only demonstrates the dynamic reversible process of lactylation but also implies a link between lactonization modification and other PTMs. For example, Class I HDAC1 to HDAC3 not only exhibit deacetylate but also play a role in delactylation of histone lysines [63]. Some studies have also indicated a potential competitive relationship between these enzymes [39], though this remains speculative and requires further experimental validation in clinical settings.

In summary, lactate metabolism not only fuels tumor growth but also contributes to gene regulation through lactylation. Both histone and non-histone lactylation are dynamically regulated by “writers” and “erasers,” integrating metabolic cues with transcriptional and post-translational control. Understanding these processes provides a comprehensive framework for exploring therapeutic interventions that target lactate production, transport, or lactylation-modifying enzymes in cancer.

## Mechanisms of lactylation in tumors

Lactylation regulates tumor biology through multiple mechanisms, influencing both cancer cell-intrinsic processes and the tumor microenvironment (TME). This section summarizes how lactylation modulates tumor cell proliferation, metabolism, and gene expression, as well as its roles in shaping immune and stromal components within the TME.

### Lactation and tumor cells

Lactylation exhibits context-dependent effects across various tumor types, influencing tumor cell proliferation, migration, invasion, and signaling pathways. This section summarizes the roles of lactylation in different cancers, highlighting both its pro-tumorigenic and, in certain contexts, growth-suppressive functions.

Lactylation varies among different tumors and plays a promoting role in tumorigenesis in some types of cancer [38, 64, 65]. For example, in digestive system malignancies, such as esophageal cancer, H3K9la activates LAMC2 transcription and protein expression to promote invasion and metastasis [66]. In gastric cancer, H3k18la increases the transcription of *vcam-1*, promotes the proliferation and migration of GC cells [67], and can promote the progression of gastric cancer through the AKT/mTOR pathway [68]. H3K18la affects a large number of NSUN2/YBX1/mTOR working along the 5C-ENO1 signaling axis, which is suggestive of the pathogenesis and classification of colorectal cancer [69]. In pancreatic ductal adenocarcinoma, H3K18la enhances *TTK* and *BUB1B* transcription and promotes tumor cell migration [70]. In hepatocellular carcinoma (HCC), non-histone CCNE2 lactonizes and promotes the proliferation, migration, and invasion of HCC cells [40]. H3K18la in renal clear cell carcinoma promotes the transcription of *PDGFRB* in endometrial carcinoma, thereby enhancing tumor proliferation and migration through the PDGFR pathway and further promoting histone lactylation to create positive feedback [71]. Although these findings are based on preclinical data, their clinical significance is yet to be fully validated. The lactylation of HIF1A in prostate cancer cells results in high PD-L1 expression, which mediates the expression of glycolytic enzymes and neuroendocrine markers [72]. In bladder cancer, H3K18la promotes the transcription of LCN2, thereby enhancing tumor cell proliferation, colony formation, and migration [73]. In female reproductive disorders, H3K18la promotes *USP39* transcription in endometrial cancer, upregulating the migration of tumor cells [74]. In cervical cancer, HIF-1 lactylation activates the transcription of *DCBLD1* and G6PD-mediated ex vivo and in vivo picropodophyllin as well as promotes the migration, invasion, and growth of cervical cancer cells [75]. In breast cancer, H3K1la upregulates the expression of LDHA, which significantly enhances the proliferation, invasion, and metastasis of breast cancer cells [76, 77]. Lactation also plays a similar role in the nervous, circulatory, and respiratory systems [71, 78, 79]. See Table 1 for details. While these findings highlight the potential role of lactylation in various cancers, clinical evidence validating these effects in human tumors remains sparse. Further clinical trials are necessary to assess the therapeutic relevance of lactylation modifications in cancer treatment. See Table 1 for details.

**Table 1:** Roles of lactylation in different tumors

Systems	Tumor	Protein	Functions	References
Digestive System	Esophageal cancer	H3K9	H3K9 Lactylation activates LAMC2 transcription under hypoxic conditions to promote tumor migration and invasion	[66]
	Gastric cancer	H3K18	H3K18 Lactylation activates VCAM-1 transcription and promotes GC cell proliferation and migration;	[68]
	Colorectal cancer	H3K18	H3K18 Lactylation activates NSUN2 gene transcription, promotes tumor development and metastasis, and participates in reprogramming of glucose metabolism	[69]
	Pancreatic cancer	H3K18	H3K18 Lactylation activates TTK and BUB1B transcription, alters the cell cycle and accelerates tumorigenesis	[70]
	Liver cancer	CCNE2	Lysine lactylation of CCNE2 promotes proliferation, migration and invasion of HCC cells	[40]
Urinary System	Clear cell carcinoma of the kidney	H3K18	H3K18 Lactylation promotes tumor proliferation and migration through positive feedback of the PDGFR pathway	[71]
	Prostate cancer	HIF1A	H3K18la upregulates the expression of neuroendocrine-related genes and promotes tumor development	[72]
	Bladder cancer	H3K18	H3K18la promotes tumor cell proliferation, colony formation and migration by enhancing LCN2 expression	[73]
Reproductive System	Endometrial cancer	H3K18	H3K18la stimulates USP39 expression to promote tumor progression	[74]
	Cervical Cancer	HIF-1	HIF-1 lactylation increases DCBLD1 expression to activate the G6PD-mediated pentose phosphate pathway and promotes cervical cancer migration, invasion, and growth	[75]
	Breast Cancer	H3K18	LDHA promotes H3K8 lactylation, induces the expression of downstream genes and LDHA itself, and promotes breast cancer cell proliferation, invasion and metastasis	[76]
Circulatory System	Acute myeloid leukemia	H3K18, H4K5, H4K8 & H4K12	H4K5 Lactylation increases PD-L1 gene expression and inhibits T cell activation	[78]
Nervous System	Glioma	H3K18La	H3K18 lactylation induces M2 macrophage polarization and promotes malignant progression	[79]
Respiratory System	Non-small cell lung cancer	H3K18	H3K18la enhances immune escape of NSCLC cells by activating the POM121/MYC/PD-L1 pathway	[80]

*Abbreviations*: *LAMC2* Laminin Gamma 2 Chain, *VCAM-1* Vascular Cell Adhesion Molecule 1, *GC* Gastric Cancer, *NSUN2* NOP2/Sun RNA Methyltransferase 2, *TTK* TTK Protein Kinase, *BUB1B* BUB1 Mitotic Checkpoint Serine/Threonine Kinase B, *CCNE2* Cyclin E2, *HCC* Hepatocellular Carcinoma, *PDGFR* Platelet-Derived Growth Factor Receptor, *H3K18la* H3K18 Lactylation, *LCN2* Lipocalin 2, *USP39* Ubiquitin Specific Peptidase 39, *HIF-1* Hypoxia-Inducible Factor 1, *DCBLD1* Discoidin, CUB and LCCL Domain Containing 1, *G6PD* Glucose-6-Phosphate Dehydrogenase, *LDHA* Lactate Dehydrogenase A

However, not all effects of lactylation promote tumor proliferation. Some studies have shown that lactate promotes tumor cell quiescence by impairing UM (Uveal Melanoma) growth through MCT1, rather than regulating the HCAR1 cascade response, which contrasts with the typical pro-tumor effect of lactylation in most other tumors [80]. This suggests that lactylation’s impact may vary significantly across different tumor types, and further clinical investigation is required to clarify its role in tumor progression.

Collectively, these findings illustrate the diverse and context-dependent roles of lactylation in tumor cells. While most evidence points to a pro-tumorigenic function, exceptions highlight the need for careful evaluation in different cancers. Understanding these mechanisms provides a foundation for exploring therapeutic strategies targeting lactylation.

### Other roles of lactylation in the TME

Beyond tumor-intrinsic effects, lactylation plays a critical role in shaping the tumor microenvironment (TME) by modulating immune cell metabolism, stromal interactions, and intercellular signaling. This section summarizes the impact of lactylation on key immune cells, macrophage polarization, and other TME components [59].

Lactic acid affects multiple immune cell types within the TME. It can influence CD8 + T cells, regulatory T (Treg) cells, natural killer (NK) cells, dendritic cells, and other immune cells by altering the acidity of the cell survival environment, among others. Excess lactic acid creates an environment favorable to tumor cell growth and promotes their invasion and metastasis [22, 81]. Lactylation can limit the value-added and other effects of T cells, mitigate T cell receptor (TCR) signaling, promote Treg cell differentiation [82], and inhibit NK cell function [83]. These effects facilitate a tumor immunosuppressive microenvironment [84]. In addition, Treg cells are integral in sustaining immunosuppressive TME [85]. Lactate enhances Treg cell stability and function, whereas lactate metabolism reduces Treg cell induction, increases antitumor immunity, and inhibits tumor growth [86, 87].

Macrophages are also present in the TME, and traditional research classifies them into two subtypes with opposing roles: M1 and M2 [88–92]. M1 macrophages exhibit proinflammatory and anticancer effects, while M2 macrophages display anti-inflammatory and protumor effects [93]. Notably, however, tumor-associated macrophages (TAMs) infiltrating the TME possess significant phenotypic complexity and are often not clear-cut M1 or M2 phenotypes, but rather exhibit mixed characteristics of both. Their polarization is dynamically regulated by microenvironmental factors [93]. Lactic acid produced by tumor cells induces the overexpression of vascular endothelial growth factor and M2-like genes, thereby driving macrophages toward an M2-like phenotype. This process accelerates tumorigenesis and cancer progression [93, 94]. Increased proprotein convertase subtilisin/kexin type 9 levels in colon cancer decreases the lactate secretion of tumor cells and reduces macrophage migration inhibitory factor (MIF) levels [19]. In contrast, MIF expression promotes the polarization of TAMs toward an M1-like phenotype, while simultaneously inhibiting their M2-related functions [91, 95]. Further studies are still needed on the effects and specific mechanisms of lactate on other substances in the TME [96].

In summary, lactylation exerts multifaceted effects in tumors, regulating both cancer cell-intrinsic processes and interactions within the tumor microenvironment. Histone and non-histone lactylation modulate gene expression, immune cell function, and stromal responses, highlighting lactylation as a central mediator of metabolic–epigenetic crosstalk. These insights provide a foundation for developing targeted strategies to manipulate lactylation for therapeutic benefit.

## Effect of tumor cell lactylation on immune cells

Histone lysine lactylation is a post-translational modification that affects gene expression regulation by disrupting the covalent link between the lactate moiety and protein lysine residues [97]. In the TME, tumor cells preferentially metabolize through glycolysis, even under aerobic conditions. Therefore, tumor cells produce a large amount of lactate during the metabolic process, reducing oxygen levels and promoting an acidic TME. Furthermore, excessive lactate accumulation can favor the establishment of an immunosuppressive environment, thereby promoting cancer cell growth and metastasis [98–100]. While these findings are supported by preclinical models, clinical studies are necessary to confirm how lactate affects gene expression and immune cell activity in human tumors. Therefore, we investigated the effects of a high-lactate environment on specific immune cells.

### Impact of a high-lactate TME on immune cells

**Fig. 3:**
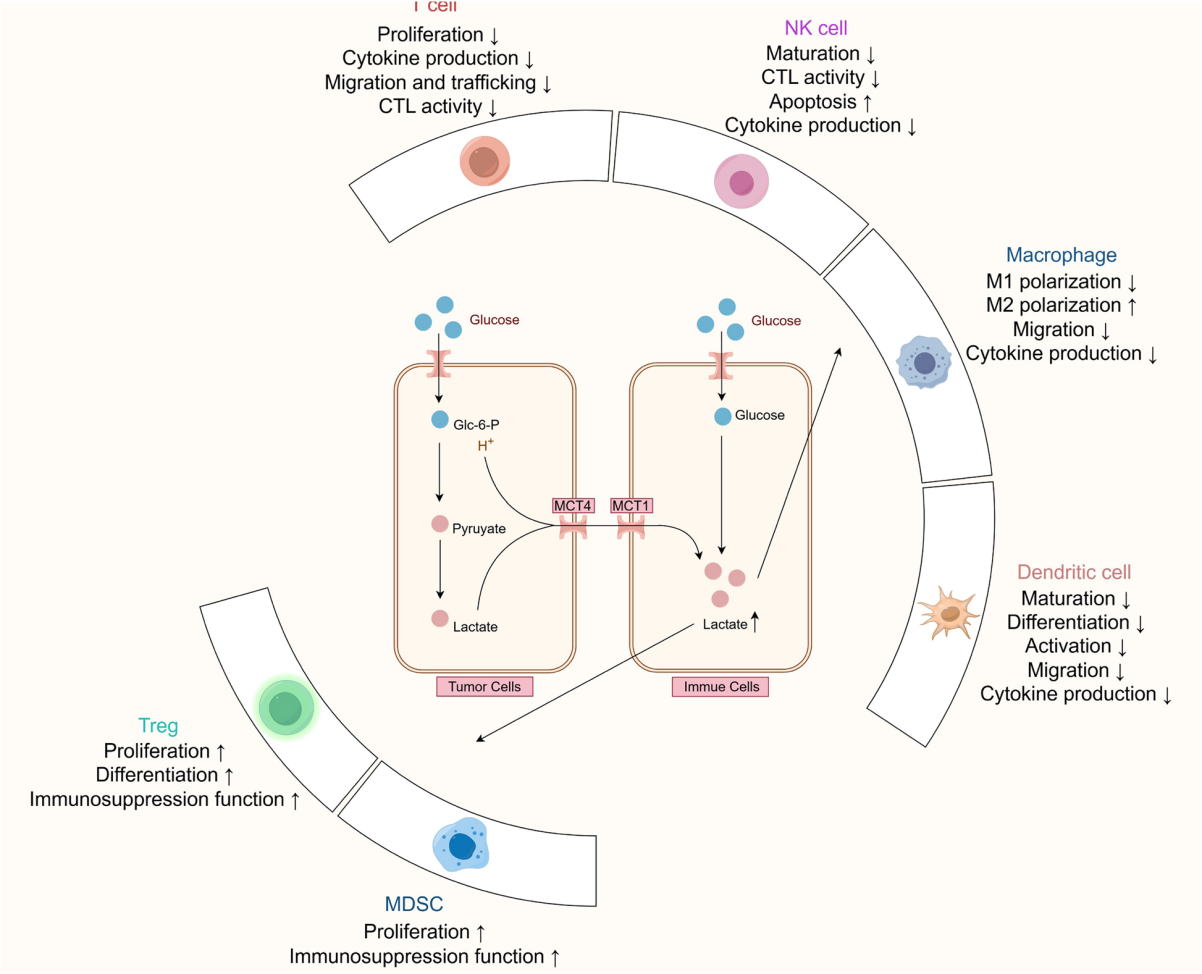
The impact of high lactate levels in the TME on various immune cells. Abbreviations: TME, tumor microenvironment

Lactate in the tumor immune microenvironment regulates the metabolism of immune cells and inhibits the proliferation and function of CD8 + T cells, NK cells, and dendritic cells, among others, thereby mediating immune escape. Furthermore, lactate can induce the polarization of M2-phenotype macrophages and promote the proliferation of Treg cells and myeloid-derived suppressor cells (MDSCs) [59, 101, 102]. These effects collectively support the establishment of an immunosuppressive TME [103]. The specific mechanism is shown in Fig. 3.

#### T cells

T cells are critical mediators of antitumor immunity, and their functions are highly sensitive to metabolic changes within the TME. This section summarizes how elevated lactate levels influence T cell viability, signaling, and effector functions, highlighting both mechanistic insights and therapeutic implications.

T cells are pluripotent stem cells derived from the bone marrow that play a critical role in the immune environment. T cells can immediately detect high levels of extracellular lactate in the TME and conduct signal transduction to maintain lactate concentration balance. However, when the lactate concentration in the TME becomes excessively high, T cell functions are inhibited. Increased lactate levels in the TME create an acidic cellular environment. The pH value of the TME is usually 6.0–6.5. This acidic environment can induce T cell apoptosis and reduce cell activity [104]. Lactate suppresses TCR signaling, inhibiting the production of effector cytokines such as interferon gamma (IFN-γ), tumor necrosis factor alpha (TNF-α), and interleukin (IL-2)[98, 105].

Lactate also impairs the functional proteins of cytotoxic T lymphocytes (CTLs) by inhibiting the phosphorylation of p38 signaling, resulting in immune suppression and reduced antitumor ability. Lactic acid also induces T cell apoptosis by reducing nicotinamide adenine dinucleotide (NAD [+]) levels [54, 98, 106]. While these findings are supported by in vitro and animal studies, clinical trials are essential to verify the relevance of lactate’s impact on T cell function in human cancers. Mechanistic studies have shown that lactate inhibits T cell proliferation through the NADH redox reaction, reducing NAD + to NADH in a high-lactate environment, thereby affecting NAD + -dependent enzymatic reactions that inhibit T cell proliferation, impair cell signaling and immune function, and promote immune escape in tumors.

Collectively, elevated lactate in the TME suppresses T cell proliferation, signaling, and effector functions, contributing to immunosuppression and tumor progression. These insights underscore the importance of targeting lactate metabolism or lactylation to restore T cell-mediated antitumor immunity and improve therapeutic outcomes.

#### NK cells

NK cells promote antitumor effects and are an important target for cancer immunotherapy. High or excessive lactate levels in the TME can induce NK cell apoptosis, thereby reducing immune cell antitumor activity [98]. Furthermore, NK cells, as potent innate immune cells, are crucial to the antitumor defense line. However, high lactate levels inhibit NKcells from producing IFN-γ and IL-4 and inhibit their survival and proliferation, thereby promoting tumor development and enhancing tumor immune escape [107, 108]. Although these observations are consistent with preclinical models, clinical studies are needed to validate the role of lactate in NK cell dysfunction in human cancers.

#### Treg cells

Regulatory T (Treg) cells are key mediators of immunosuppression in the tumor microenvironment (TME), and their functions are strongly influenced by high lactate levels. This section summarizes how lactylation and lactate metabolism regulate Treg differentiation, proliferation, and survival, highlighting implications for tumor immune escape and therapy.

Treg cells act as a major mediator of immunosuppressive effects under high-lactate conditions in the tumor microenvironment (TME) [54]. The TME promotes the differentiation and proliferation of Treg cells by increasing the expression of key transcription factors FOXP3 and MCT1. This process has been observed in various tumor types, where the active recruitment of Treg cells contributes to immune suppression and immune escape [109, 110]. FOXP3 is a key transcription factor that controls Treg cell development and function. High FOXP3 expression can inhibit glycolysis, enhance oxidative phosphorylation, and increase NAD (+) oxidation to promote Treg cell metabolism, thereby enhancing Treg cell adaptibilty and survival in acidic and high-lactate TMEs [110, 111].

MCT1 is a transmembrane protein that transports lactate and H^+^ across the cell membrane. MCT1 expression is highly significant in tumor cells, as it initiates abundant lactate production in the TME [88]. Therefore, MCT1-mediated lactate influx, H^+^ efflux, and cellular metabolism are crucial for the survival of Treg cells in the TME [46]. Numerous Treg cells in the TME are a key indicator of the immune escape of tumor cells. While research into targeting Treg cells to suppress tumor immune escape is limited, studies focusing on high lactate levels in the TME could become a crucial area of exploration for regulating Treg cell function in cancer therapy [88, 112].

Collectively, the abundance and metabolic adaptation of Treg cells in high-lactate TMEs contribute to tumor immune escape. Targeting lactate metabolism or Treg-specific pathways represents a promising approach to modulate immunosuppression and enhance antitumor immunity, warranting further investigation [88, 112].

#### Dendritic cells

Dendritic cells are very important antigen-presenting cells that efficiently absorb, process, and transmit antigen information to CD8 + T cells [98]. The high lactate levels in the TME can inhibit the differentiation and signal transduction of dendritic cells, as well as induce the inactivation of cytokines secreted by dendritic cells. Lactic acid can also stimulate IL-10 production to inhibit the activation of dendritic cells. In addition, lactate can promote the production of IFN-α by plasma cell-like dendritic cells (pDCs) through two mechanisms. The first mechanism is through the binding of lactate to GPR81 receptors on the surface of pDCs, altering intracellular calcium ion concentrations and inhibiting IFN-γ production [113]. However, high extracellular lactate concentration inhibits the export of lactate from dendritic cells, leading to lactate accumulation in the cytoplasm of pDCs and disrupting the glycolytic process. Therefore, lactate inhibits glycolysis and IFN-γ production by controlling the energy production pathway of pDCs [114, 115]. While these findings are supported by preclinical research, clinical studies evaluating lactate's impact on dendritic cell function in human cancers are still limited, and further investigation is needed to confirm these effects in vivo.

Collectively, high lactate concentrations in the TME impair dendritic cell differentiation, cytokine secretion, and glycolytic activity, thereby reducing their capacity to activate T cells and promote antitumor immunity. These findings underscore the potential of targeting lactate metabolism or lactylation to restore DC function and enhance immune responses in cancer therapy, though further in vivo and clinical validation is needed.

#### Macrophages

Macrophage polarization refers to the process by which macrophages adopt different functional states, primarily classified into two phenotypes: M1 and M2 [116]. M1 macrophages are proinflammatory and cytotoxic, typically activated by lipopolysaccharide (LPS) and interferon-gamma (IFN-γ). In contrast, M2 macrophages exhibit anti-inflammatory properties and play roles in tissue repair, and are activated by cytokines such as IL-4 and IL-13. Recent studies have highlighted the role of lactylation, a post-translational modification, in regulating macrophage function. Lactylation involves the addition of lactate groups to proteins, altering their stability and activity [117, 118].

Macrophages are key immune cells involved in phagocytosis and pathogen clearance, and their polarization significantly influences immune responses. While M2 macrophages are generally associated with anti-inflammatory effects and repair functions, tumor-associated macrophages (TAMs) present a more complex behavior. TAMs, which infiltrate the tumor microenvironment (TME), initially exhibit an immune-promoting phenotype, similar to M1 macrophages, in the early stages of tumor development. However, as the tumor progresses, environmental factors such as hypoxia and high lactate levels in the TME induce a shift in TAM polarization toward an immunosuppressive M2-like phenotype [100, 119, 120]. This transition contributes to immune escape and metastasis [121, 122].

Lactate, a key metabolic product in the TME, plays a central role in driving TAM polarization toward the M2 phenotype. High lactate levels not only inhibit the secretion of pro-inflammatory cytokines, such as IL-10 and TGF-β, by M2 macrophages, but also suppress the cytotoxic activity of tumor-infiltrating lymphocytes (TILs) and promote the differentiation of regulatory T (Treg) cells. Additionally, lactate directly affects macrophage metabolism by inhibiting the glycolytic enzyme PFK-1 (phosphofructokinase-1), leading to a reduction in glycolytic flux and diminished immune function in monocytes. Furthermore, lactate acts as a signaling molecule to promote the polarization of M2-like macrophages in myeloid-derived suppressor cells (MDSCs), further enhancing the immunosuppressive environment of the TME [111, 123, 124].

Although these findings are supported by preclinical studies, the clinical relevance of lactate-induced macrophage polarization in human cancers remains to be fully explored. Clinical studies are required to validate these mechanisms in vivo and assess their therapeutic potential.

#### MDSCs

Myeloid-derived suppressor cells (MDSCs) are a heterogeneous population of immunosuppressive cells that play a critical role in inhibiting the activity of both innate immune cells, such as NK cells, and adaptive immune cells, including CD4 + and CD8 + T cells. These cells contribute significantly to immune suppression in the tumor microenvironment (TME) by performing various immunosuppressive functions. Studies have shown that the expression profiles of glycolytic genes and MDSC-related genes are often correlated, and both are associated with poorer prognosis in cancer patients. Elevated glycolysis and lactate concentrations are known to promote MDSC differentiation and function, contributing to the establishment of an immunosuppressive environment [98, 125].

High lactate levels in the TME not only stimulate the proliferation of MDSCs but also impair the cytotoxic activity of immune cells like NK cells, which further promotes immune escape and tumor progression. Through these mechanisms, lactate helps to establish an immune-suppressive TME, facilitating tumor growth and metastasis. The increased presence and activity of MDSCs under high lactate conditions amplify these immunosuppressive effects, further promoting tumor development [98, 125].

While these observations are supported by preclinical studies, clinical data linking lactate-induced MDSC differentiation and immune suppression in human cancers are still limited. Further clinical trials are needed to assess the therapeutic potential of targeting MDSCs in the context of the lactate-rich TME.

### Autolactylation of immune cells

Autolactylation in immune cells represents a potential mechanism linking metabolism with immune regulation. This section summarizes current evidence on HMGB1 lactylation in macrophages, histone lactylation in T cells, and the broader implications for immune-cell differentiation and tumor immunity.

HMGB1 is a nuclear protein that triggers inflammatory reactions by activating macrophages. Existing studies have shown that elevated lactate levels can promote HMGB1 lactylation in macrophages during sepsis, suggesting that lactylation participates in inflammation-related metabolic regulation [50, 126]. In addition, histones in macrophages isolated from mouse melanoma and lung tumor models have been found to undergo lactylation, and increased histone lactylation is positively associated with the protumor phenotype of M2 macrophages, indicating a potential link between histone lactylation and macrophage-driven tumor progression [50].

The relationship between T cell lactylation and tumor progression is crucial in tumor immunity. As discussed previously, Treg cells contribute to an immunosuppressive microenvironment, and lactate accumulation may influence their function. Current studies suggest that histone lactylation may represent a regulatory mechanism relevant to inflammatory diseases and tumor immunity; however, direct evidence in human tumors remains limited, and most findings are derived from preclinical models [101, 127, 128]. In diseases such as sepsis, cancer, chronic inflammation, and autoimmune diseases, lactate produced through aerobic glycolysis induces immunosuppression in local tissues. Enhanced glycolysis can increase the availability of biosynthetic substrates that support inflammatory protein synthesis after immune activation. However, aside from the reported macrophage lactylation events, additional forms of immune-cell self-lactylation have not been clearly identified, and evidence regarding how lactylation directly affects immune-cell differentiation remains insufficient. Current studies hypothesize that tumor-cell–derived lactate may influence immune cells through lactylation-related pathways, but this hypothesis lacks direct experimental confirmation and requires further investigation [101, 111, 128].

## Application of lactylation targeting in cancer therapy

**Fig. 4:**
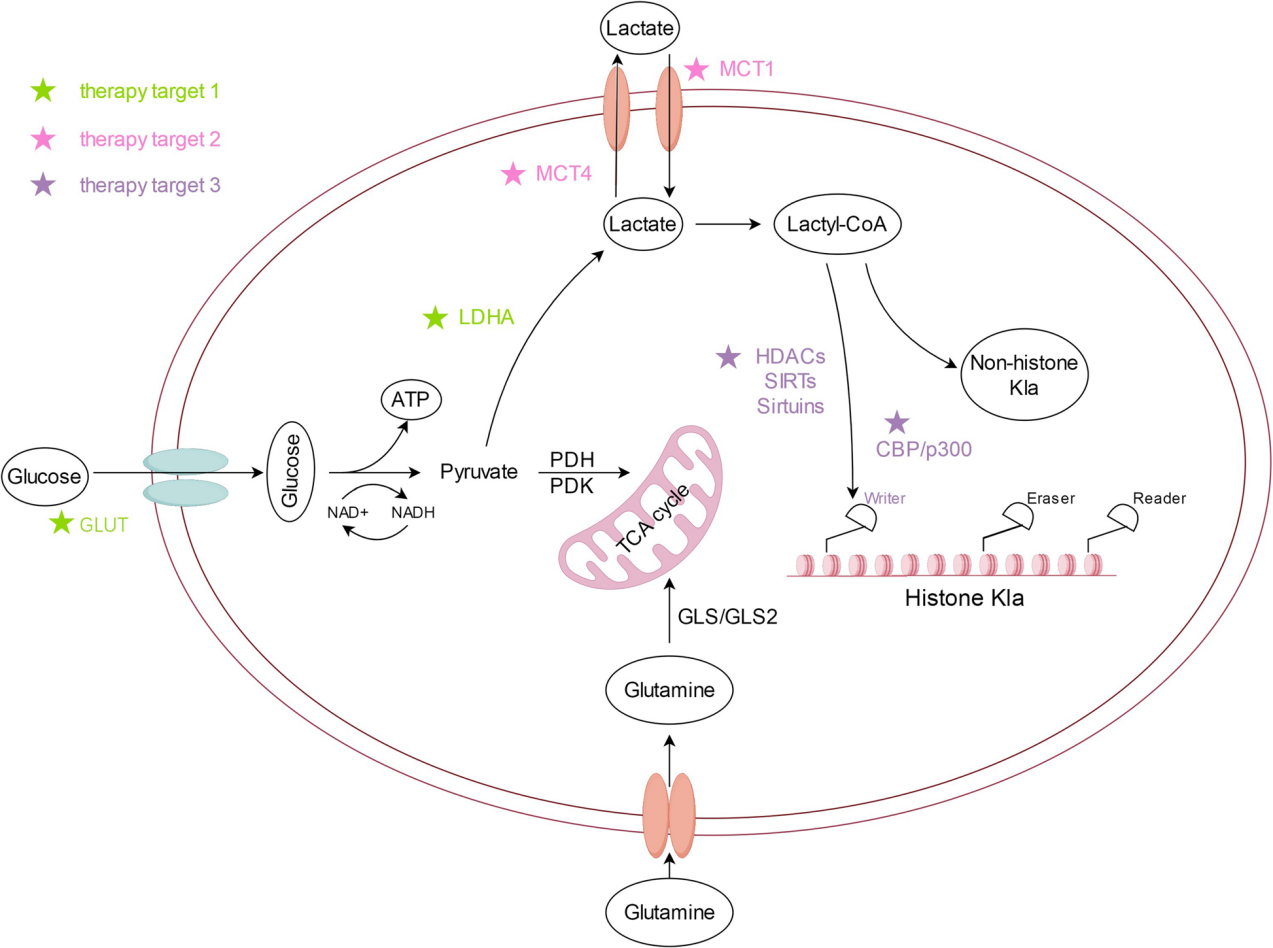
Three targeted pathways and their mechanisms for targeted lactation therapy for tumors (targeted lactation therapy for tumors is an innovative treatment method that uses specific drugs to target molecules or biological processes related to tumor cells and delivers the drugs to the tumor site through the lactation pathway, aiming to kill tumor cells effectively while minimizing damage to normal tissues.). Therapeutic target 1 represents lactate dehydrogenase A and glucose transporters, which mainly target metabolic pathways. Therapeutic target 2 represents monocarboxylate transporters (MCT)1 and MCT4, which mainly target lactate transport. Therapeutic target 3 regulates the balance between the writer and eraser during lactation. Abbreviations: MCT1/4, monocarboxylate transporter 1/4

Lactylation directly impacts gene expression and is essential for cancer progression [1–4, 13, 19, 21, 22, 97, 100, 101, 104, 111, 113, 118, 119, 122, 124, 125, 129–143]. Lactate, a byproduct of cancer cell metabolism, not only augments the tumor energy supply but also can modulate the tumor immune microenvironment by inhibiting CTL recruitment and inducing NK cell apoptosis, as demonstrated in cell and animal models [138, 141]. Kla is associated with cancer progression, angiogenesis, and drug resistance and may influence various cancer therapies [97, 104, 111, 113, 114, 124, 125, 130, 133–136]. Lactylation has become a new target for cancer therapy because of its effects on metabolic, transcriptional, and epigenetic mechanisms [13, 100, 101, 103, 125, 130, 134–138, 144]. Targeting Kla-related enzymes or modulating Kla modifications is being explored in preclinical studies as a potential therapeutic strategy, but clinical evidence is currently limited [101, 111]. The specific mechanism is shown in Fig. 4.

### Targeting the regulation of lactylation

In addition to being an essential source of cellular energy, lactate is also essential for signal transduction and the control of gene expression [13, 144–148]. Current evidence demonstrating these functions primarily comes from preclinical studies, including cell-based and animal models. Disrupting lactate homeostasis is now considered a viable approach for cancer therapy, as reducing lactate production can indirectly reduce lactylation, thereby providing a feasible mechanism for tumor treatment [103, 140, 149–152]. However, clinical evidence directly targeting lactate metabolism or lactylation in cancer patients remains limited, and further clinical investigation is required to validate these therapeutic concepts.

#### Reducing lactate production and transport

**Fig. 5:**
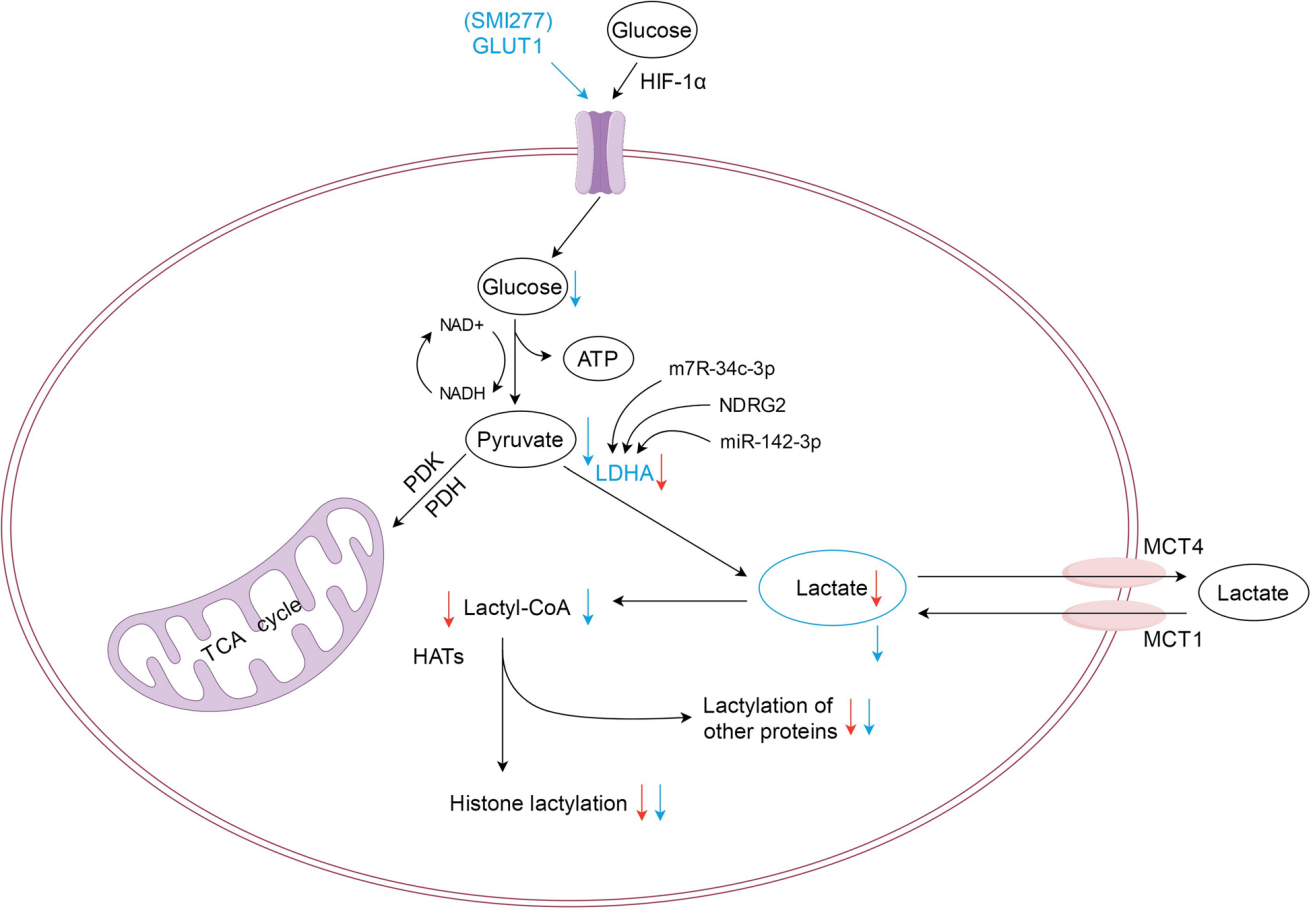
Diagram demonstrating the inhibition of lactate dehydrogenase A (LDHA) expression and targeting glucose transporter 1 (GLUT1) to affect glucose transport can effectively reduce lactate production and further reduce the lactylation of tumor cells to achieve therapeutic purposes. Abbreviations: LDHA, lactate dehydrogenase A; GLUT1, glucose transporter 1

**Fig. 6:**
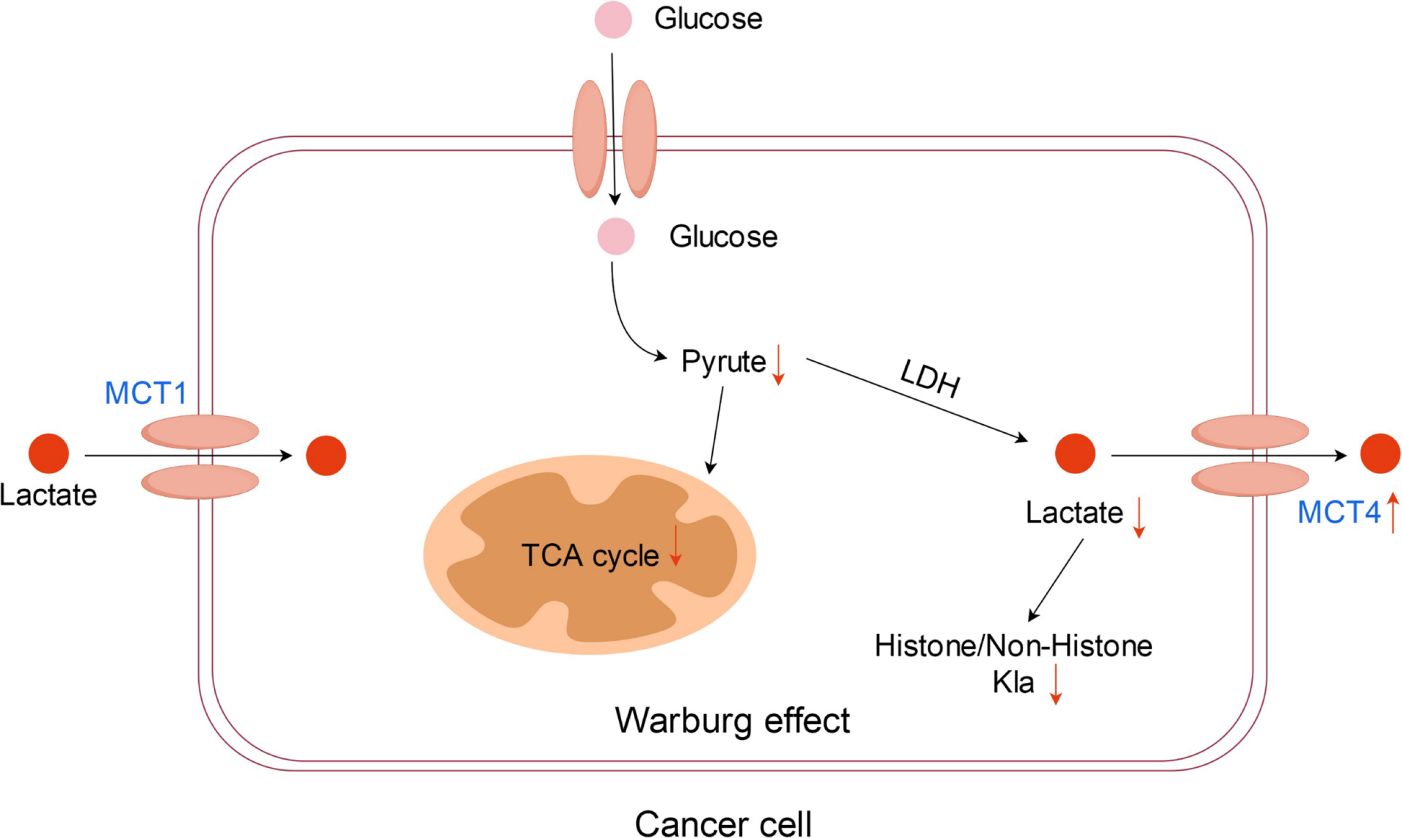
Diagram demonstrating the targeted effect on lactate transport was mainly directed at monocarboxylate transporter (MCT4). Increasing MCT4 expression can facilitate extracellular lactate transport, suppress lactate accumulation in tumor cells, alter the tumor microenvironment, and inhibit lactylation. Abbreviations: MCT4, monocarboxylate transporter 4

Extracellular lactate has been shown in preclinical studies to influence histone lysine lactylation (Kla), whereas glycolysis may modulate it in the opposite direction [1, 153]. Histone Kla levels are significantly affected by endogenous lactate production, suggesting a potential strategy for targeting lactylation in cancer therapy; however, these concepts are primarily based on cell and animal models and require clinical validation [1, 153]. Lactate production may be linked to increased glucose uptake, elevated glycolytic enzyme expression and activity, and impaired mitochondrial function, as well as enhanced lactate production, accumulation, and release, which is facilitated by MCT1 and MCT4 [142, 145]. The specific mechanisms are shown in Figs. 5 and 6.

A critical approach to curbing lactate production lies in inhibiting lactate dehydrogenase (LDH) expression. LDH (lactate dehydrogenase), especially LDHA, is a key enzyme in glycolysis and lactate metabolism that performs the conversion of pyruvate into lactate [154]. The overexpression of LDHA in various tumor cells has been observed in preclinical studies and represents a potential therapeutic target [55, 100, 138, 141, 155]. Regulating the activities of lactate dehydrogenase and pyruvate dehydrogenase can lower intracellular lactate levels and provide insights into the reduction of endogenous lactate production [39]. However, these observations are primarily preclinical. LDH inhibitors have been shown in cell- and animal-based studies to downregulate lactate metabolism and inhibit lactylation by blocking downstream lactylation pathways [1, 150, 156, 157]. The efficacy of LDH inhibitors may depend on specific isoforms and microenvironmental factors, such as tumor type and metabolic context, based on preclinical evidence [140]. LDHA inhibitors can be classified into free enzyme-binding inhibitors, NADH-competitive LDHA inhibitors, pyruvate-competitive LDHA inhibitors, dual pyruvate and NADH-competitive LDHA inhibitors, and other unknown types of LDHA inhibitors [132, 141]. Inhibiting the expression of upstream genes regulating LDH, including *HIF1A*, *HSF1*, oncogene *MYC*, and *CREB1*, has been explored in preclinical studies as a potential strategy to modulate lactate metabolism [138, 158].

Overall, inhibiting LDHA or its upstream regulators offers a promising approach to reduce lactate accumulation and lactylation in tumors. Preclinical evidence supports the potential of these interventions to impair tumor growth, reprogram the TME, and enhance the efficacy of combination therapies, although clinical validation is still required.

Another key strategy for interfering with lactate dynamics focuses on targeting MCT expression. The overexpression of MCTs has been observed in various cancer cells and is associated with increased lactate production in preclinical studies [132, 140, 159]. MCTs are essential for lactate transfer and are potential key players in the co-production of lactate-dependent metabolism, based on preclinical evidence [23, 113, 114, 125, 160]. Four transporters in the MCT family (MCT1 to MCT4) have been identified [23, 159]. Targeting MCTs has been proposed as a potential strategy to modulate lactate-dependent metabolism, although most data come from cell- and animal-based studies. Known MCT inhibitors, such as organic mercurials, stilbene disulfonates, α-cyano-4-hydroxycinnamate, and other drugs, have shown efficacy in preclinical models of cancers, such as gastric cancer, breast cancer, and Burkitt’s lymphoma [100, 138, 140, 160–162]. The specific mechanisms are shown in Fig. 6.

Furthermore, MCT1 dysfunction has been shown in preclinical studies to impair pyruvate homeostasis without necessarily reducing lactate flux [145, 163, 164]. Many malignancies are accompanied by high levels of MCT4 expression, especially in hypoxic regions, with rapidly growing tumor masses. The loss of MCT4 function impairs the invasiveness of tumor cells expressing MCT4 and disrupts the metabolic symbiosis between various cell populations, making MCT4 a potential therapeutic target for many tumors [140]. Moreover, 7-amino-coumarin derivatives have been shown in preclinical studies to interfere with mitochondrial pyruvate transport via MCT1 and MCT4, promoting intracellular pyruvate accumulation and inhibiting extracellular lactate uptake [140, 150, 165].

In summary, MCTs are critical regulators of lactate metabolism in tumors, and targeting their expression or function offers a promising approach to reduce lactate accumulation, disrupt lactylation, and impair tumor metabolic adaptation.

Collectively, targeting lactate production and transport represents a promising strategy to modulate lactylation in tumors. Preclinical evidence suggests that inhibiting key enzymes such as LDHA, regulating upstream transcription factors (e.g., HIF1A, MYC), or blocking lactate transporters (MCT1/MCT4) can decrease intracellular lactate, impair lactylation, and disrupt tumor metabolic adaptation. While most findings are derived from cell and animal models, these approaches provide a mechanistic basis for future clinical evaluation and the development of combination strategies aimed at enhancing antitumor efficacy.

#### Metabolic regulation

Glucose is the primary source of intracellular lactate, and preclinical studies suggest that it can promote lactate production and histone Kla levels in a dose-dependent manner [1, 166]. In addition to glycolysis, glutamine degradation contributes to lactate production in cancer cells [13, 167]. In the cytoplasm, glucose undergoes classical catalytic reactions to produce pyruvate, which is then directly reduced to lactate by LDH instead of being oxidized in the mitochondria. The interplay between pyruvate dehydrogenase and glycolysis significantly influences lactate levels, as demonstrated in preclinical experiments [13, 168]. The specific mechanism is shown in Fig. 5.

Lactylation and glycolysis are closely linked, and modulating lactylation may influence glycolytic activity, which is being explored as a potential therapeutic approach in preclinical studies. Increased protein lactylation levels in tumors are linked to glucose transporter 1 (GLUT1) overexpression, which also facilitates the polarization of TAMs, based on preclinical evidence [13, 169, 170].

Furthermore, lactylation modifies the metabolic status of cells, which influences not only the phenotype of cancer cells but also the development of therapeutic resistance, although most evidence is preclinical [13]. Numb/Parkin-mediated mitochondrial adaptation, regulated by histone lactylation, is a potential target for cancer treatment in preclinical studies since it can distinguish the vital metabolic processes of cancer cells [13, 71]. The main targets in glycolysis and glutamine metabolism pathways include LDHA, MCTs, and GLUT. The roles of LDHA and MCT have already been described. Targeting histone Kla using GLUT1 inhibitors is another therapeutic approach, although off-target effects on other metabolic processes remain a concern [132].

### Disrupting the balance between writers and erasers

**Fig. 7:**
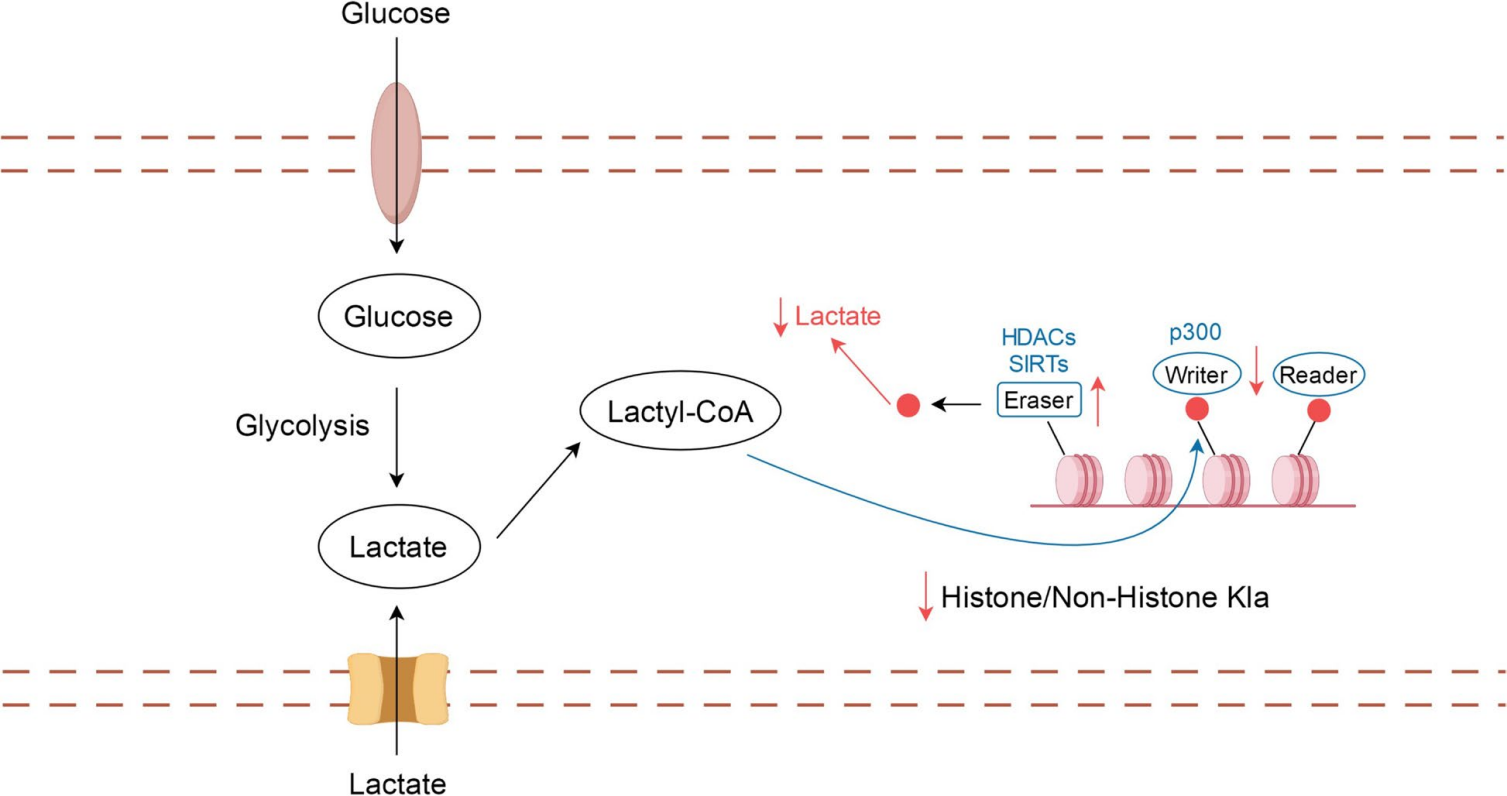
Diagram showing an imbalance between writer and eraser. Both the inhibition of writer expression and increased eraser expression can be used to target lactylation, especially histone lactylation and thus facilitate tumor treatment

Based on the mechanisms of acetylation writers (e.g., KAT), erasers (e.g., KDAC), and readers (e.g., bromodomain) in Kla, preclinical studies suggest that disrupting the balance between writers and erasers present potential targets for disrupting lactylation in cancer treatment [101, 138]. Therefore, reducing the expression of writers and increasing the expression of erasers is a potentially effective method for treating cancer, although this approach is primarily based on cell- and animal-based models. The specific mechanism is shown in Fig. 7.

p300 and SIRT3 serve as writers and erasers, respectively, and play key roles in tumor growth, proliferation, metastasis, and invasion in preclinical studies [100, 171]. SIRT3 specifically targets cyclin E2 (CCNE2) as a substrate for inhibiting lysine lactylation (Kla), based on preclinical evidence [40, 100]. Furthermore, acetylation erasers such as the HDAC family, are important drug targets involved in post-translational modifications. HDAC inhibitors (HDACi) have demonstrated efficacy in preclinical and clinical studies of hematological malignancies [39, 101, 138, 172–174].

p300 was identified as a histone lactylation “writer,” representing the first discovery of an enzyme mediating this modification [171]. p300/CREB-binding protein (CBP) is an acetylase targeting more than two-thirds of acetylation sites and is closely related to malignant tumors in preclinical studies [171, 175]. p300 is essential for IFN-γ–induced PD-L1 expression and is a potentially useful target for overcoming adaptive resistance produced through the PD-1/PD-L1 pathway. Synthetic CBP/p300 inhibitors have shown potential as an effective treatment for hematological malignancies in preclinical studies [100, 176]. Furthermore, reducing p300 levels has been observed to nonspecifically decrease histone Kla levels in preclinical studies [132].

In summary, the dynamic balance between lactylation “writers” (e.g., p300) and “erasers” (e.g., SIRT3, HDACs) represents a key regulatory axis in tumor lactylation and metabolism. Preclinical evidence indicates that modulating this balance—by reducing writer activity or enhancing eraser activity—can decrease lactylation, inhibit tumor growth, and sensitize tumors to therapy. Although most studies are based on cell and animal models, targeting writers and erasers provides a mechanistic framework for developing novel lactylation-targeted interventions in cancer treatment.

### Unidentified target compounds

In addition to the pathways with the well-defined targets mentioned previously, many compounds currently act on Kla and influence the development of diseases like cancer by regulating histone Kla levels; however, their precise molecular targets remain unclear. These include evodiamine, Royal Jelly Acid, and demethylzeylasteral, all of which have been studied primarily in cell- and animal-based models [73, 132, 151, 177].

In summary, therapeutic strategies targeting lactylation in cancer encompass multiple approaches, including inhibition of lactate production and transport, metabolic modulation, and disruption of the balance between “writers” and “erasers.” While several preclinical studies demonstrate promising efficacy, many potential targets remain unidentified, and clinical validation is limited. Together, these insights highlight both the opportunities and challenges in translating lactylation-targeted interventions into effective cancer therapies.

## Lactylation targeting for combination therapy

Single-target lactylation therapy has demonstrated efficacy in preclinical cancer models, but combining it with other therapies, particularly immunotherapy, remains a potential strategy that requires further validation [101]. Lactylation is highly dependent on lactate availability, which is primarily regulated by lactate metabolism, including lactate production via LDH and lactate transport via MCTs, based on preclinical studies [12, 100]. Therefore, targeting lactate metabolism may indirectly inhibit lactylation by reducing its substrate supply, while direct modulation of lactylation (e.g., inhibiting lactyltransferases or activating delactylases) could provide a more precise approach in preclinical models [12, 113, 178]. Targeting lactylation in stem cells is also a potential direction for cancer research, although evidence is currently limited to preclinical studies.

### Combination of lactylation targeting and immunotherapy

Metabolic targeting in cancer faces complex challenges since metabolic therapies may affect immune system function, as suggested by preclinical studies [79]. Targeting lactylation in combination with immune checkpoint inhibitors (ICIs) offers potential solutions for overcoming this challenge, although current evidence is primarily based on preclinical models [178]. This combination may modulate tumor metabolism while potentially influencing antitumor immunity through lactylation-mediated epigenetic or post-translational modifications, but its efficacy and safety in humans remain to be established.

#### ICI and lactate–lacylation targeting combination therapy

ICIs, such as CTLA-4 and PD-1 inhibitors, have shown significant and lasting therapeutic responses in certain cancers in clinical studies [113, 179–185]. However, many patients exhibit poor responses to ICI treatment, which may be related to the TME [113, 184, 186]. In a highly glycolytic TME, preclinical studies suggest that the activation of PD-1 + Treg cells may cause treatment failure when PD-1 alone is targeted [113, 187, 188]. Combining glycolysis and mTOR inhibitors exerts synergistic therapeutic effects in preclinical models of hematological malignancies [113, 189, 190]. Therefore, metabolic modulators, especially lactylation targeting combined with ICI therapy, holds a great therapeutic potential, although current evidence is largely preclinical [100, 101, 113, 118, 191]. The specific mechanism is shown in Fig. 1.

In tumors with high glycolytic activity, preclinical studies show that Treg cells express higher PD-1 levels than effector T cells [100, 105, 187]. In low-glucose conditions, Treg cells increase PD-1 expression after taking up lactate via MCT1, whereas effector T cells decrease PD-1 expression, based on in vitro and animal models. Furthermore, lactate stimulates PD-L1 expression in neutrophils and macrophages, thereby contributing to immune evasion [100, 192]. Preclinical studies have also confirmed that combining ICIs with lactate metabolism inhibitors—a form of indirect lactylation targeting—can exert synergistic effects. For example, the MCT1 inhibitor AZD3965 blocks lactate uptake by Treg cells and tumor cells in preclinical models, leading to reduced intracellular lactate levels and subsequent inhibition of lactylation. This intervention has been shown to decrease PD-1 expression on Treg cells and PD-L1 expression on myeloid cells, while restoring effector T cell function, thereby enhancing antitumor immunity in preclinical studies [54, 100, 153, 164].

In preclinical models, inhibiting MCT4—which mediates lactate export from tumor cells—reduces TME lactate accumulation and lactylation levels, resulting in increased CD8 + T cell activation, reduced TME acidification, and enhanced chemokine (e.g., CXCL10) secretion, all of which may improve the efficacy of anti-PD-1 therapy [113, 116, 153]. Targeting LDHA, a key enzyme for lactate production, has also been shown in preclinical studies to indirectly inhibit lactylation. LDHA inhibition reduces lactate generation in tumor cells, leading to decreased global lactylation—including histone and non-histone lactylation—within the TME [193]. Specifically, blocking LDHA has been reported to reduce lactylation of the PD-L1 promoter histone H3K18, suppressing PD-L1 expression on tumor cells and myeloid cells, and thereby enhancing the antitumor immune response in combination with anti-PD-1 treatment in preclinical studies [100, 113, 194]. Although the application of LDHA inhibitors in vivo still needs optimization, they hold significant research value as a potential pathway for regulating PD-L1 expression and enhancing the efficiency of anti-PD-1 therapy [100, 113, 194]. Collectively, these findings suggest that combining indirect lactylation targeting (via LDH/MCT inhibitors) or direct lactylation targeting (e.g., lactyltransferase inhibitors) with immunotherapy may be an effective approach for overcoming lactylation-mediated immune evasion, but clinical validation is still needed [49, 100].

#### Combination of targeting lactylation with chimeric antigen receptor T cell (CAR-T) therapy

CAR-T therapy has achieved remarkable clinical results in hematological malignancies but generally shows limited efficacy in solid tumors, which is thought to be partly due to the immunosuppressive TME and restricted CAR-T cell persistence [8–10, 113, 195]. Preclinical studies suggest that lactylation may contribute to these limitations: excessive lactate in the TME can induce lactylation of CAR-T cells and tumor cells, potentially promoting CAR-T cell exhaustion and Treg cell differentiation (i.e., CAR-Treg cells) [12]. Therefore, targeting lactylation—either directly or indirectly—may improve CAR-T therapy efficacy by modulating metabolism and lactylation in both tumor cells and CAR-T cells, as suggested by preclinical models. For instance, LDHA inhibition, as a form of indirect lactylation targeting, has been shown in preclinical studies to reprogram glucose metabolism in cancer stem cells and reduce TME lactate levels, thereby inhibiting lactylation [11].

In a mouse glioblastoma model (GBM), Sun et al. [12] found that the LDHA inhibitor and CAR-T combination therapy altered the glucose metabolism of cancer stem cells, mitigated the immunosuppressive TME, and decreased the presence of CAR–Treg cells within the tumor. Mechanistically, in these preclinical models, reduced H3K18 lactylation in cancer stem cells suppressed the transcription of immunosuppressive factors (e.g., IL-10), while in CAR-T cells, it inhibited the differentiation of CAR-Treg cells and reduced adenosine production in the TME [12, 113, 196, 197]. However, these findings are limited to preclinical studies, and further research is needed to confirm their applicability in human CAR-T therapy. Furthermore, some researchers propose that lactate may exert a protective effect on antitumor immunity, indicating that the incorporation of lactate during T cell expansion could enhance the effectiveness of CAR-T therapy, although this remains speculative and requires experimental validation [11].

#### Lactylation-targeting vaccines in cancer therapy

Cancer vaccines are a form of active immunotherapy. However, they still demonstrate insufficient immunogenicity, and the immune evasion mechanisms of tumor tissues hinder their effectiveness [113, 198]. Lactate can enhance the efficacy of cancer vaccines in preclinical studies. Feng et al. [11] discovered that MC38 mice treated with lactate exhibited stronger antitumor effects than those treated with glucose. Mechanistically, in these preclinical models, lactate inhibits HDAC activity, leading to increased histone lactylation in CD8 + T cells and dendritic cells (DCs) [113, 199]. Lactylation of DC-specific transcription factors (e.g., IRF8) promotes DC maturation and aggregation, whereas lactylation of CD8 + T cell exhaustion-related histones (e.g., H3K23la) may delay T cell exhaustion—these effects were observed independently of pH changes. These findings indicate that lactate may improve the efficacy of tumor vaccines by promoting dendritic cell maturation and lowering immunosuppression in the TME [101, 113]. However, these effects have so far only been demonstrated in preclinical studies, and further research is required to determine whether similar mechanisms operate in human patients. Future studies could explore combining cancer vaccines with targeted lactylation modulators (e.g., low-dose MCT inhibitors or specific lactyltransferase activators) to optimize lactylation levels and potentially enhance immunogenicity.

Collectively, preclinical evidence indicates that combining lactylation-targeted strategies with immunotherapy—including immune checkpoint inhibitors (ICIs), CAR-T therapy, and cancer vaccines—can enhance antitumor immunity by modulating TME metabolism, reducing immunosuppressive lactylation, and promoting effector T cell function. Direct lactylation targeting (e.g., lactyltransferase inhibitors) and indirect targeting (e.g., LDHA or MCT1/MCT4 inhibition) offer complementary approaches to overcome lactate-mediated immune evasion. While these findings are predominantly preclinical, they provide a mechanistic rationale for future clinical evaluation of combination therapies aiming to optimize immune responses and improve therapeutic outcomes.

### Combination of lactylation targeting with other drugs

Research on the combination of lactylation targeting with other drugs is limited [200, 201]. Preclinical studies suggest that combining LDHA inhibitors (indirect lactylation targeting) with biguanides may enhance the efficacy of melanoma treatment by synergistically reducing lactate production and lactylation [140].

Lactate accumulation is a hallmark of solid tumors. In preclinical models, TIP60-mediated lactylation of NBS1 at the K388 site promotes the development of the MRN complex, thereby facilitating DNA repair and increasing cancer cell resistance to genotoxic agents [202]. Therefore, targeting lactate metabolism (to reduce the substrate for lactylation) or directly inhibiting NBS1 K388 lactylation may block this DNA repair pathway and potentially overcome chemoresistance, although these strategies remain largely preclinical [202]. Notably, the clinically available LDHA inhibitor stiripentol shows significant synergy with chemotherapeutic agents, such as cisplatin and IR, to effectively suppress cancer cell survival in preclinical studies. These findings suggest that combining lactylation-targeting agents (either indirectly via LDH/MCT inhibitors or directly via site-specific lactylation inhibitors) with chemotherapeutic agents could be a promising strategy to improve therapeutic outcomes, but further clinical studies are needed to confirm efficacy in patients [202].

In summary, combining lactylation-targeted strategies with immunotherapy or other pharmacological interventions offers a promising avenue to enhance anticancer efficacy. Preclinical studies indicate that such combination approaches can potentiate immune checkpoint inhibitors, CAR-T therapy, and therapeutic vaccines, while addressing tumor heterogeneity and resistance. Nevertheless, optimal combinations, dosing, and patient stratification remain to be determined, highlighting the need for further mechanistic studies and clinical validation.

## Conclusion and future direction

Lactylation has emerged as a crucial link between tumor metabolism and gene regulation, integrating cellular energy status with transcriptional and post-translational modifications. Current evidence highlights its role in modulating tumor cell proliferation, metastasis, immune evasion, and therapy resistance. Histone and non-histone lactylation influence both intrinsic tumor processes and extrinsic TME interactions, particularly through effects on Treg cells, macrophages, dendritic cells, and NK cells. These findings underscore lactylation as a central metabolic-epigenetic hub with significant implications for cancer biology and therapy.

Despite these advances, several critical questions remain unresolved. The specificity of lactylation relative to other post-translational modifications in regulating gene expression is still unclear. Additionally, the dependence of different tumor types or contexts on lactylation has not been systematically characterized. Reliable biomarkers, such as lactylated histones in patient biopsies, are urgently needed to guide therapeutic interventions and monitor responses. Moreover, while preclinical studies suggest promising strategies targeting lactylation—directly via writers, erasers, and readers or indirectly via LDH/MCT modulation—robust clinical validation remains limited.

Therapeutically, combining lactylation-targeted strategies with immunotherapy or chemotherapy holds significant promise. Preclinical models indicate that inhibition of lactate production, transport, or specific lactylation sites can enhance the efficacy of ICIs, CAR-T therapy, and chemotherapeutic agents. Nonetheless, the complex interplay between tumor metabolism, immune regulation, and lactylation necessitates careful evaluation of potential off-target effects, context-specific responses, and patient stratification.

Looking forward, future research should prioritize the integration of multi-omics approaches, patient-derived samples, and mechanistic studies to delineate context-dependent functions of lactylation across tumor types and immune cell populations. Particular attention should be given to clarifying its causal roles in Treg cells, tumor-associated macrophages, and adoptive immune cell therapies, as well as to the development of site-specific or substrate-selective inhibitors. Addressing these open questions will not only advance our understanding of metabolic-epigenetic crosstalk but also inform innovative treatments that leverage lactylation modulation for improved clinical outcomes.

## Data Availability

Not applicable.
